# Culture supplement extracted from rice bran for better serum-free culture

**DOI:** 10.1186/1753-6561-7-S6-P104

**Published:** 2013-12-04

**Authors:** Satoshi Terada, Satoko Moriyama, Ken Fukumoto, Yui Okada, Rinaka Yamauchi, Yoko Suzuki, Masayuki Taniguchi, Shigeru Moriyama, Takuo Tsuno

**Affiliations:** 1 Department of Applied Chemistry and Biotechnology, University of Fukui, Fukui, 910-8507, Japan; 2 Niigata University, Niigata, 950-2102, Japan; 3 Tsuno Food Industrial Co., Ltd, Katsuragi-cho, Wakayama, 649-7122, Japan

## Introduction

In mammalian cell culture, fetal bovine serum (FBS) and proteins including albumin (BSA) have been extensively added to culture media as growth factor. But mammal-derived factors are potent source of various infections such as abnormal prion and viruses, and so alternative supplement is eagerly required. The alternative must be chemically defined or obtained from plant, as well as should be produced in commercial quantities and stably supplied.

As an alternative supplement, we focused on rice bran extract (RBE), by-product of milling in the production of refined white rice, because rice bran contains abundant nutrients and proteins [1] as well as antioxidants [2] and because rice is cultivated plant, indicative of huge and stable supply.

## Materials and methods

### Preparation of RBE

RBE was extracted in alkaline solution and then precipitated with acid. The precipitate was freeze-dried.

### Effect of RBE on the culture of various cell lines

Mitogenic activity of RBE was evaluated using cell lines. Cells were cultured in ASF104 medium with or without RBE for several days. Then viable cell densities were counted by trypan-blue method and concentration of MoAb was measured by ELISA.

### Effect of RBE on the culture of MSC

Mesenchymal stem cells (MSCs) were isolated from male Wistar rats and expanded in purchased serum-free medium or conventional medium containing FBS. The expanded cells were transferred to differentiation medium into bone. The differentiated cells to bone were readily stained. Triplicated culture.

### Effect of RBE on the culture of pancreatic islets

Pancreatic islets were obtained from male Lewis rats and cultured in RPMI medium supplemented with RBE or FBS for eight days.

## Results and discussion

### Effect of RBE on the culture of various cell lines

On growth and MoAb production of hybridoma in serum-free medium, desired effects of RBE were observed and the effect was superior to BSA.

Similarly, serum-free culture of CHO-DP12 added with RBE exhibited increased cell growth and production.

Growth of HepG2 and HeLa cells in the serum-free medium was also improved.

Together all, RBE had mitogenic activity on various cell lines.

### Effect of RBE on the culture of MSC

As primary cells, MSCs from Wistar rat were expanded in serum-free medium with RBE or without and then the medium was changed into osteoblast-inducing medium. While MSCs expanded in the serum-free medium lost it, the cells expanded in the presence of RBE retained the potency, suggesting that RBE contains physiologically active substances maintaining potency of differentiation during *ex vivo *serum-free culture.

### Effect of RBE on the culture of pancreatic islets

Pancreatic islets, isolated from Lewis rats, were also tested in the presence of RBE. While islets died out by one week in basal medium, islets successfully survived in the presence of RB. This result supports that RBE could alternate FBS in islets culture.

**Figure 1:**
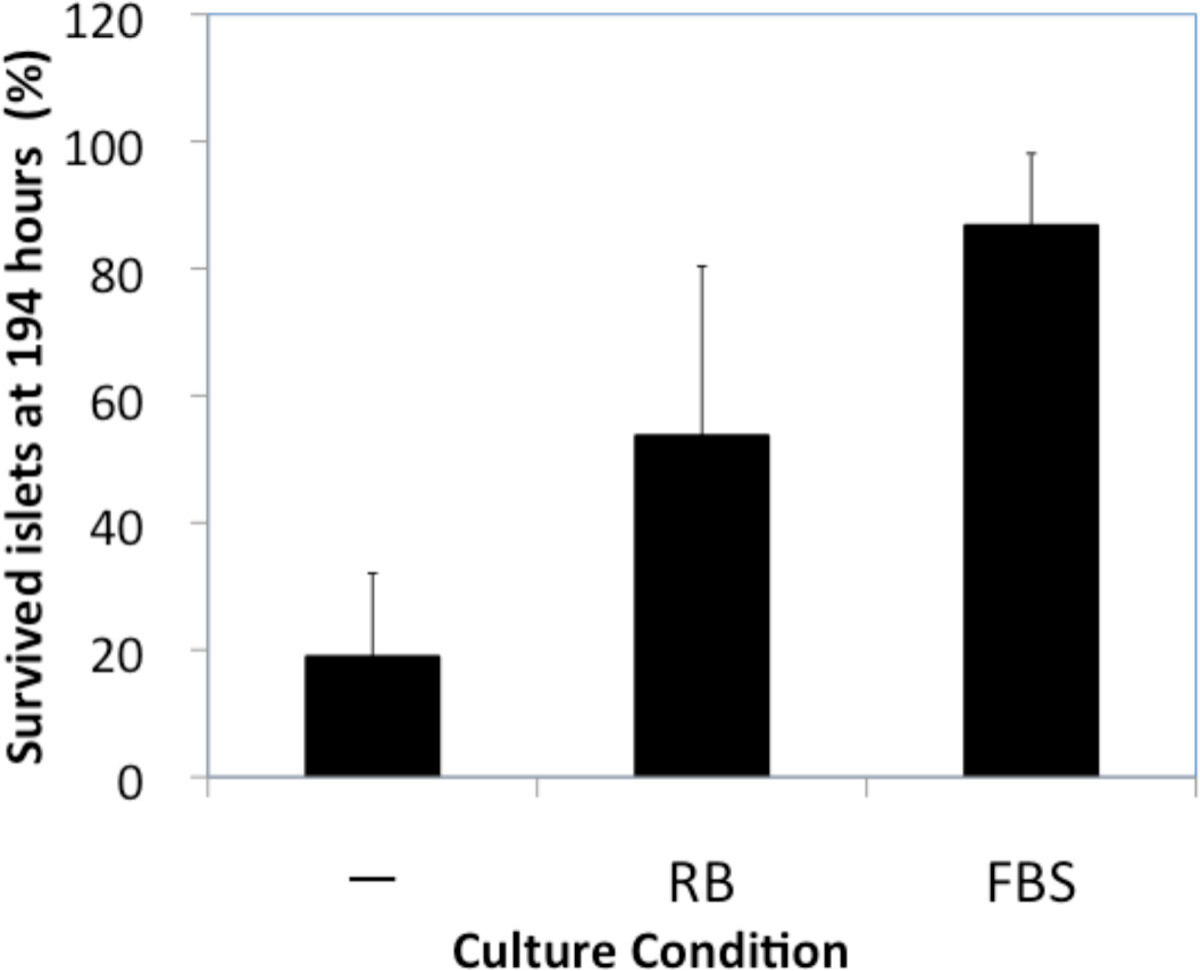
**Effect of RBE on Serum-free Culture of Islets**. Islets were cultured in RPMI 1640 medium in the presence of RBE or FBS as positive control.

## Conclusion

RBE successfully improved the serum-free culture of four cell lines including hybridoma, CHO, HepG2 and HeLa, as well as primary culture of MSCs and pancreatic islets. These results indicate that RBE would be useful as culture supplement in serum-free media.

## References

[B1] Adebiyi AP Adebiyi AO Hasegawa Y Ogawa T Muramoto K Isolation and characterization of protein fractions from deoiled rice bran European Food Research and Technology 2008 7

[B2] Adebiyi AP Adebiyi AO Yamashita J Ogawa T Muramoto K Purification and characterization of antioxidative peptides derived from rice bran protein hydrolysates European Food Research and Technology 2008 7

